# Pseudoaneurysm rupture presenting as bleeding from the cannulation site in a paediatric patient with dilated cardiomyopathy and congenital skin lesions requiring EXCOR^®^ Paediatric ventricular assist device: a case report

**DOI:** 10.1093/ehjcr/ytaa108

**Published:** 2020-05-15

**Authors:** Yuji Doi, Nao Hamamoto, Masaki Osaki, Motonori Ishido

**Affiliations:** y1 Department of Cardiology, Shizuoka Children’s Hospital, 860 Urushiyama, Aoiku, Shizuoka City, Shizuoka 420-8660, Japan; y2 Cardiac Care Unit, Shizuoka Children’s Hospital, 860 Urushiyama, Aoiku, Shizuoka City, Shizuoka 420-8660, Japan; y3 Department of Cardiovascular Surgery, Shizuoka Children’s Hospital, 860 Urushiyama, Aoiku, Shizuoka City, Shizuoka 420-8660, Japan

**Keywords:** Left ventricular assist device, Pseudoaneurysm, EXCOR^®^, Paediatric, Case report

## Abstract

**Background:**

EXCOR^®^ Paediatric is used worldwide as a bridge-to-transplant treatment. It provides improved patient stability during the waiting period compared with previous ventricular assist device (VAD). However, investigations into complications which may occur among the paediatric population during long waiting periods are still sparse.

**Case summary:**

We describe the case of a 7-year-old girl who presented with severe heart failure due to dilated cardiomyopathy. She also had a skin lesion which appeared soon after birth. She had received an EXCOR^®^ implant and was waiting for heart transplant. Her skin lesion worsened after implantation and she suffered recurrent infections. Multiple bleeding episodes from the cannulation site occurred; therefore, surgical exploration of the bleeding was performed. She passed away during the procedure due to massive bleeding caused by rupture of a pseudoaneurysm caused by blood-stream infection.

**Discussion:**

Patients with skin disease may be at increased risk of infection when on a VAD. Infections that occur during VAD therapy may cause serious complications such as pseudoaneurysm. The possibility of pseudoaneurysm should be considered when bleeding occurs in a patient on VAD.

**Learning points**

- Patients with skin lesions may have an increased risk of infections while on EXCOR^®^; consequently, these patients will require careful monitoring to prevent the development of infections.
- Pseudoaneurysm is a rare but fatal complication in patients with an implanted EXCOR^®^ device. Therefore, contrast-enhanced computed tomography should be considered before surgical exploration for unexplained bleeding from the cannulation site.

## Introduction

The EXCOR^®^ Paediatric is a ventricular assist device (VAD) that is used widely as a bridge-to-transplant therapy. It can support circulation in patients of any age for long periods with good results.^1^^,^^2^ A major complication of implantation of the EXCOR^®^ Paediatric that is related to morbidity is neurological injury, mainly due to ischaemic stroke.^3^ Other complications, such as infection around the cannulation site, occur quite frequently; other rare complications may occur in prolonged implantation. Rupture of pseudoaneurysm due to infection is a rare, but fatal, complication. Reports of this complication among patients on EXCOR^®^ Paediatric are scarce.

## Timeline

**Table:** 

5 months prior to admission	Electrocardiogram abnormality detected on school checkup
1 month prior to admission	Started to have heart failure symptoms
Day 1	Admission due to severe heart failure
Day 31	EXCOR^®^ Paediatric implantation
Day 767	Sepsis due to methicillin-resistant *Staphylococcus aureus*
Day 768	First bleeding from the cannulation site of return flow cannula
Day 775	Second bleeding from the same site
Day 781	Demise due to bleeding caused by pseudoaneurysm rupture

## Case presentation

**Figure 1:**
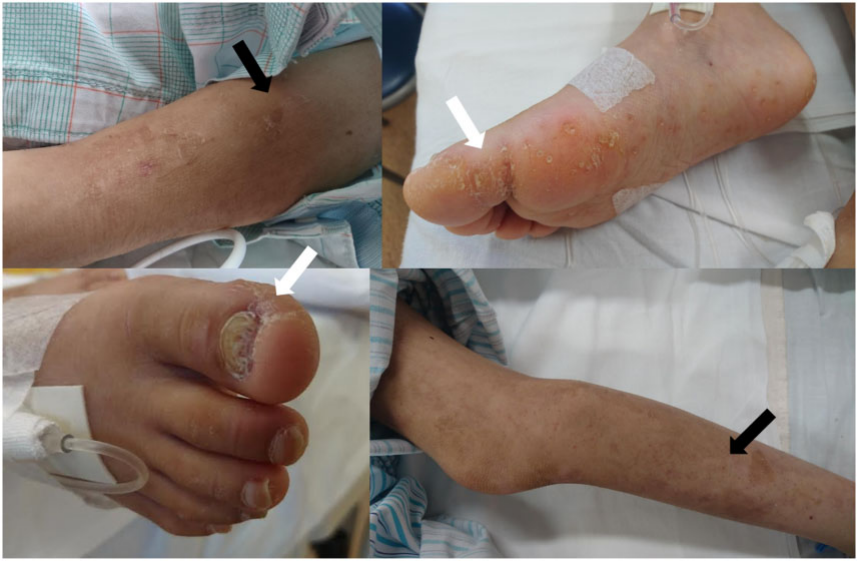
Photographs of congenital skin lesions seen in the present case. Prominent hyperkeratosis (white arrow) and bullous formation (black arrow) are mainly observed on the extremities.

**Figure 2:**
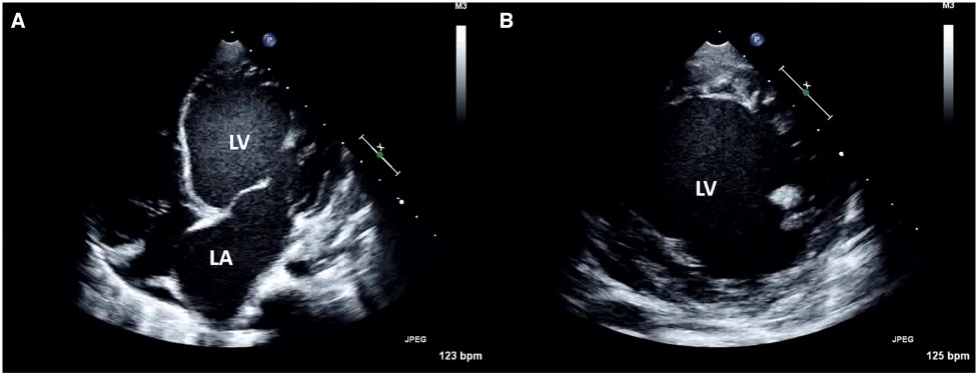
Images of echocardiography showing an extremely dilated left ventricle and atrium in (*A*) four-chamber view and (*B*) short-axis view.

**Figure 3:**
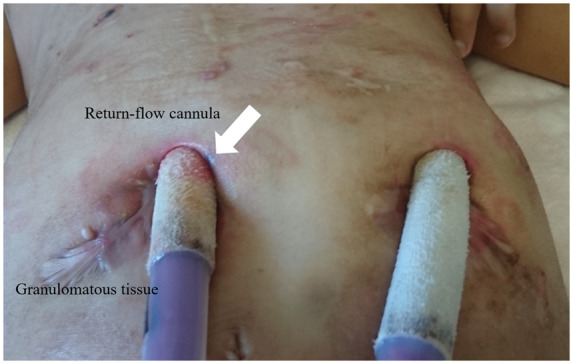
Photograph of the skin around the cannulation site. The cannula on the right side (white arrow) is the return flow cannula, which exhibited a worse condition with continuous granulomatous tissue formation.

**Figure 4:**
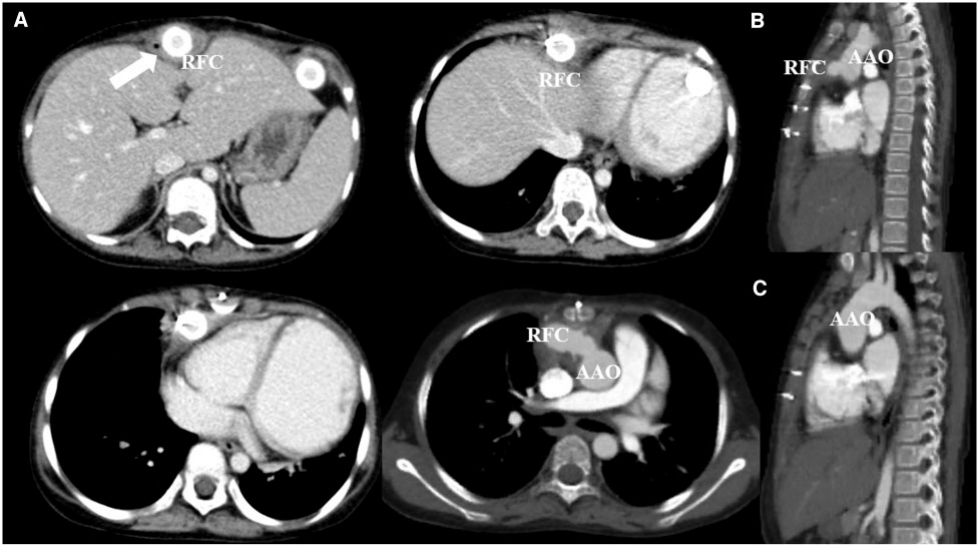
Contrast-enhanced computed tomography performed on Day 729. (*A*) Axial view showing fluid retention around the cannulation site (white arrow), which is suggestive of infection. No signs of pseudoaneurysm are observed at this time. (*B* and *C*) The sagittal view is shown. The ascending aorta is intact.

We present the case of a 7-year-old girl who was referred to our hospital with severe symptoms of heart failure. An abnormal Q wave was noted during a school checkup, but the patient did not receive medical attention. She had severe fatigue and dyspnoea at the time of admission. Gallop rhythm was heard and coolness of extremities was prominent. She also had hyperkeratosis and a bullous skin lesion of unknown cause which appeared soon after birth (*Figure 1*). Echocardiography revealed a dilated left ventricle and ejection fraction was <20% with Teichholz method (*Figure 2*). Chest X-ray showed massive cardiomegaly and congestion. We diagnosed her with idiopathic dilated cardiomyopathy. We administered diuretics, dobutamine, milrinone, and human atrial natriuretic peptide, yet her heart function worsened soon after admission. She required extracorporeal membrane oxygenation support on Day 25 but did not recover thereafter. We decided to implant EXCOR^®^ as a bridge-to-transplant therapy. We did not consider the skin lesions as a contraindication because they were located on the extremities. After implantation of EXCOR^®^, genetic testing detected a homozygous loss-of-function mutation of calpastatin (*CAST*). Both parents were found to be heterozygous for the mutation, and because other examinations, including skin biopsy and mitochondrial enzyme activity, did not reveal any abnormality, this was thought to be the cause of her skin lesions. Skin condition around the return flow cannula (RFC) worsened gradually, and granulomatous tissue formation occurred (*Figure 3*). She occasionally experienced fever, which we treated as sepsis although a positive blood culture was never observed. At nearly 2 years after admission, she started to experience intermittent fever which required antibiotics. There was no vegetation on transthoracic echocardiography, and contrast-enhanced computed tomography (CECT) performed on Day 729 showed fluid retention along the RFC (*Figure 4*). We continued debridement and antibiotic administration when required. On Day 767, she had another episode of high fever, with a positive blood culture of methicillin-resistant *Staphylococcus aureus* (MRSA). We administered vancomycin and daptomycin, and the subsequent blood culture was negative for MRSA. However, massive bleeding from the cannulation site of the RFC occurred on Days 768 and 775. Because the bleeding stopped by compression, cardiovascular surgeons considered that exploration of the cannulation site could be diagnostic and practical; yet we could not determine the origin of bleeding by the exploration, the bleeding was considered to be caused by neoplastic vessel along the RFC, and did not perform further images or exams. On Day 781, she had another episode of massive bleeding. At this time, we decided to perform surgical exploration with the intention of exchanging the whole system if needed. We did not perform CECT before moving to the operation room because the bleeding did not stop. When the sternum was opened, a tremendous amount of bleeding occurred instantly due to a ruptured pseudoaneurysm at the cannulation site of the ascending aorta. Despite all medical efforts, she collapsed into asystole and then to ventricular fibrillation. She passed away during surgical exploration.

## Discussion

**Figure 5:**
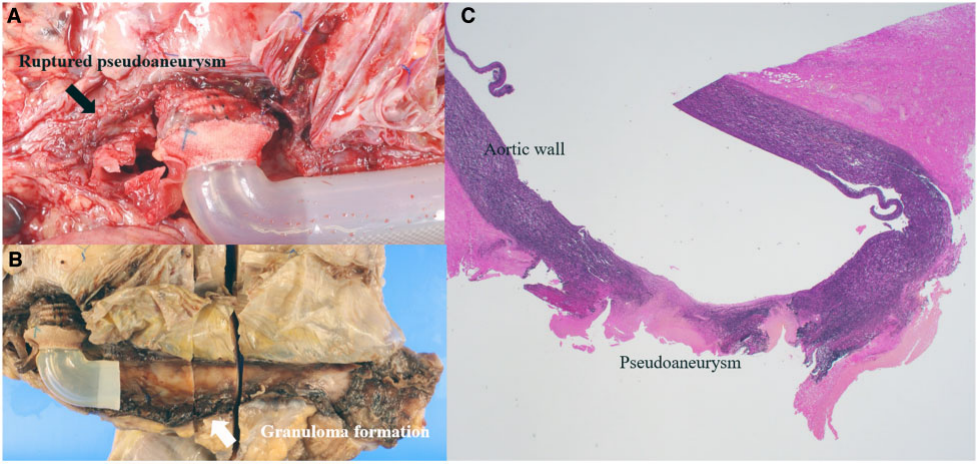
(*A*) Ruptured pseudoaneurysm adjacent to the cannulation site of the ascending aorta (black arrow). (*B*) Autopsy specimen after formalin preservation showing inflammatory changes and granuloma formation that progress from the cannulation site to the pseudoaneurysm in the ascending aorta (white arrows).

The EXCOR^®^ is now widely used as an established circulatory assist device for children with severe heart failure^1^ and was approved for use in Japan in 2016. Although there have been some reports of pseudoaneurysm in adult VAD patients,^4^^,^^5^ there have been very few studies on this in the paediatric population. To the best of our knowledge, there is only one report of pseudoaneurysm which occurred after removal of the device.^6^ In the present case, pseudoaneurysm occurred while the patient was on the EXCOR^®^, and unfortunately, this is the first report of death due to ruptured pseudoaneurysm in this context. Formation of pseudoaneurysms may be caused by graft contact with the sternal wire^5^^,^^6^ or by infection.^7^ We believe that the pseudoaneurysm in the present case was formed due to blood-stream infection which occurred because the skin barrier was compromised. Histopathology showed inflammatory changes of the tissue with neutrophil infiltration and granuloma formation from the cannulation site all the way to the pseudoaneurysm, which formed in the ascending aorta, disrupting the integrity of the aortic wall (*Figure 5*). We speculate that the bleeding from the pseudoaneurysm presented as bleeding from the cannulation site, as these sites were spatially connected. The bleeding stopped in the first instance because the patient had cardiomegaly even after EXCOR^®^ implantation, and the bleeding site was compressed by the sternum from the inside. The use of imaging modalities such as CECT should be considered whenever bleeding from the cannulation site occurs. The mortality rate for surgical repair of pseudoaneurysm may be high,^8^ and repair of ruptured pseudoaneurysm combined with the need to place another cannula for EXCOR^®^ may contribute to the development of neurological complications following surgery. In the present case, surgery under cardiopulmonary bypass via femoral access, deep hypothermia and selective cerebral perfusion may have been an option to minimize neurological damage.^9^ However, this method may not have maintained a neurological state which would qualify for heart transplant. Endovascular treatment might be another option, and stenting the pseudoaneurysm including the VAD circuit or embolizing the pseudoaneurysm with coils has also been reported.^6^

Skin lesions such as atopic dermatitis can be a risk factor for skin infection, and may even cause sepsis.^10^ These lesions are also considered a risk factor for infective endocarditis,^11^ and this concept may be adaptable for patients with similar skin lesions. Pseudoaneurysm formation, on the other hand, is often related to infections and could be due to direct extension or haematogenous seeding.^8^ Drive line infection (DLI) is a frequent late complication in VAD patients and could develop into sepsis.^12^ While DLI is not necessarily related to mortality in adult patients with left ventricular assist device,^13^ nor in paediatric populations with long-term use of EXCOR^®^,^14^ we assume that patients with skin barrier compromise are at a higher risk of infection, and possibly concomitant pseudoaneurysm formation. Here, we report the case of a patient with a loss-of-function mutation of *CAST*, which encodes the endogenous protease inhibitor calpastatin. This protein plays an important role in the protease inhibitor balance in epidermal homeostasis,^15^ and the loss-of-function mutation causes an autosomal-recessive condition characterized by generalized peeling skin, leuconychia, acral punctate keratoses, cheilitis, and knuckle pads,^15^ as seen in the present case. Skin lesions are not a contraindication for VAD implantation, but the high risk of infection and difficulties involved in clinical management should be considered, especially in the long term.

## Conclusions

Skin lesions are not a contraindication for VAD implantation, but they are associated with higher risk of infection, including DLI or sepsis, especially in regard to long implantation periods. Infections may lead to pseudoaneurysm formation and rupture, which may present as bleeding from the cannulation site, and therefore warrants careful evaluation and management.

## Lead author biography

**Figure:**
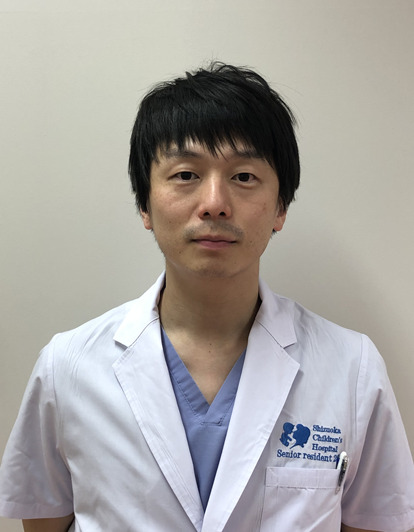


Yuji Doi graduated from Osaka University School of Medicine in 2011. After 2 years of junior residency, he started general paediatric residency at Shizuoka Children’s Hospital in 2013. He then moved on to Cardiology fellowship at Shizuoka Children’s Hospital from 2016 to 2019. He is currently working at Kurashiki Central Hospital as a paediatric cardiologist. His has interest particularly in electrophysiology.

## Supplementary material

Supplementary material is available at *European Heart Journal - Case Reports* online.

## Supplementary Material

ytaa108_Supplementary_Slide_Set
