# Supplementary material for: Pseudoaneurysm rupture presenting as bleeding from the cannulation site in a paediatric patient with dilated cardiomyopathy and congenital skin lesions requiring EXCOR® Paediatric ventricular assist device: a case report
Source: Eur Heart J Case Rep. 2020 May 15;4(3):1–6. doi: 10.1093/ehjcr/ytaa108 (PMC7319848; doi:10.1093/ehjcr/ytaa108)
Supplement: ytaa108_Supplementary_Slide_Set [file ytaa108_supplementary_slide_set.pptx]

## Slide 1
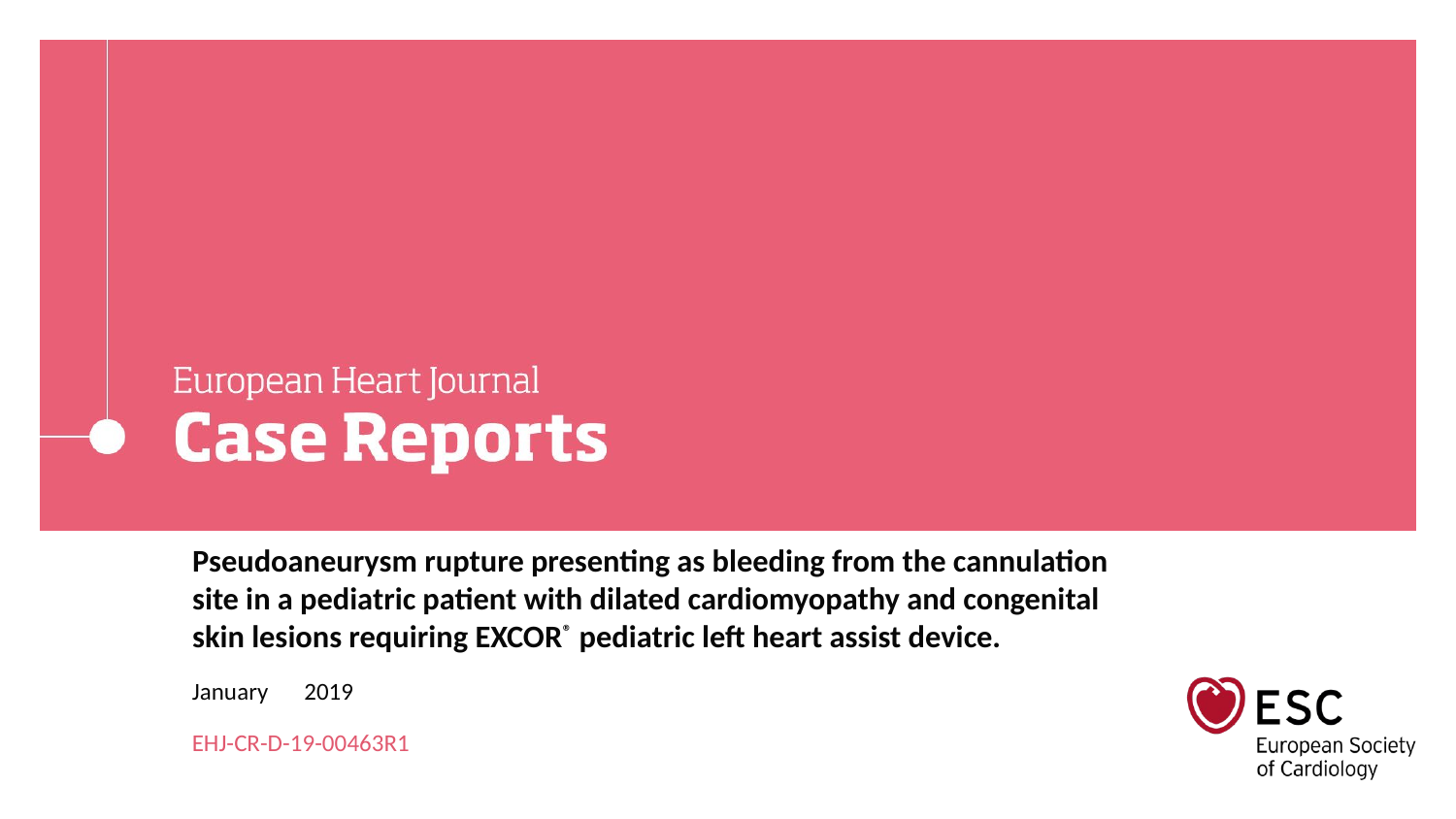

# Pseudoaneurysm rupture presenting as bleeding from the cannulation site in a pediatric patient with dilated cardiomyopathy and congenital skin lesions requiring EXCOR® pediatric left heart assist device.
January　2019
EHJ-CR-D-19-00463R1

## Slide 2
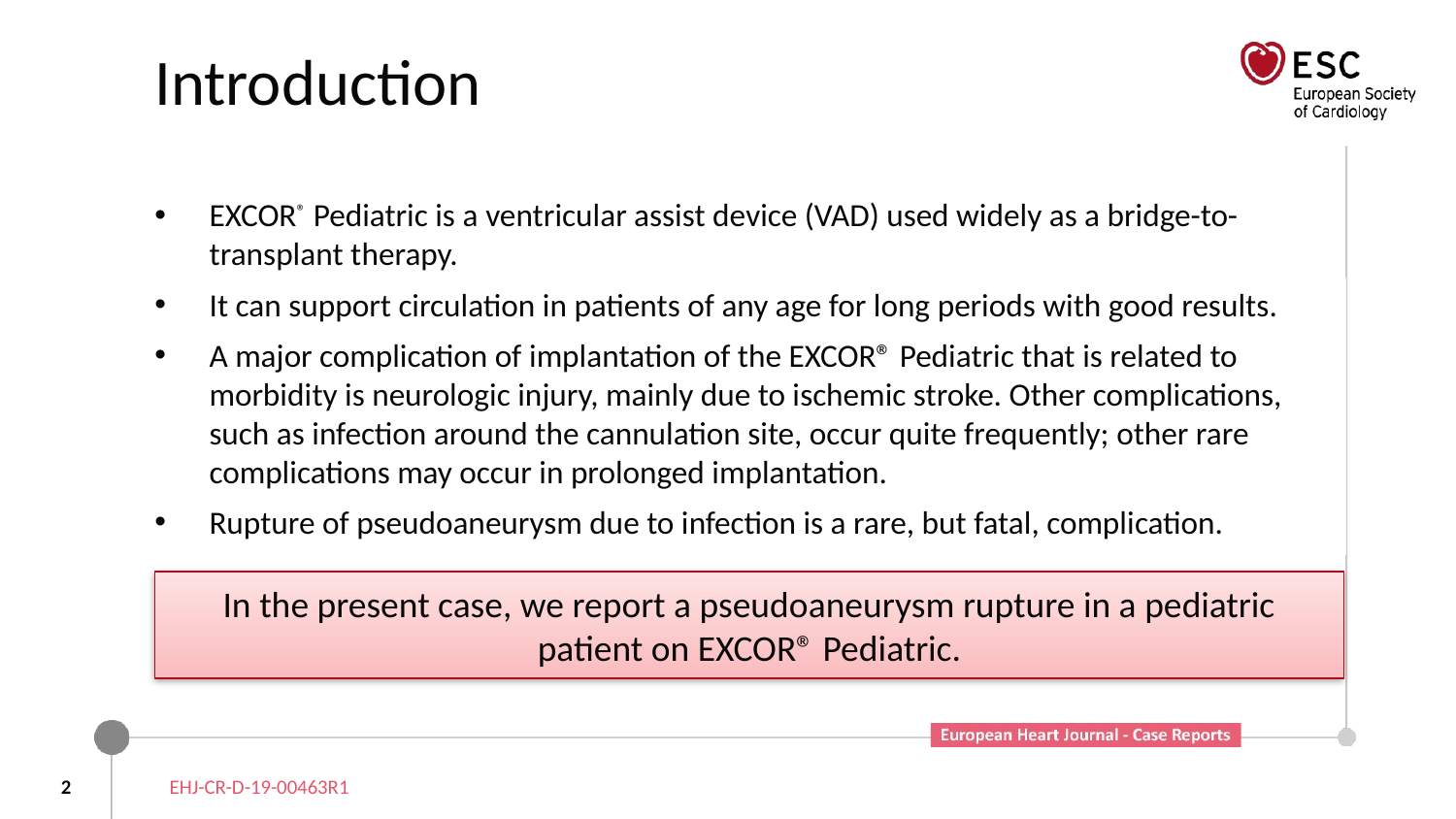

# Introduction
EXCOR® Pediatric is a ventricular assist device (VAD) used widely as a bridge-to-transplant therapy.
It can support circulation in patients of any age for long periods with good results.
A major complication of implantation of the EXCOR® Pediatric that is related to morbidity is neurologic injury, mainly due to ischemic stroke. Other complications, such as infection around the cannulation site, occur quite frequently; other rare complications may occur in prolonged implantation.
Rupture of pseudoaneurysm due to infection is a rare, but fatal, complication.
In the present case, we report a pseudoaneurysm rupture in a pediatric patient on EXCOR® Pediatric.
2
EHJ-CR-D-19-00463R1

## Slide 3
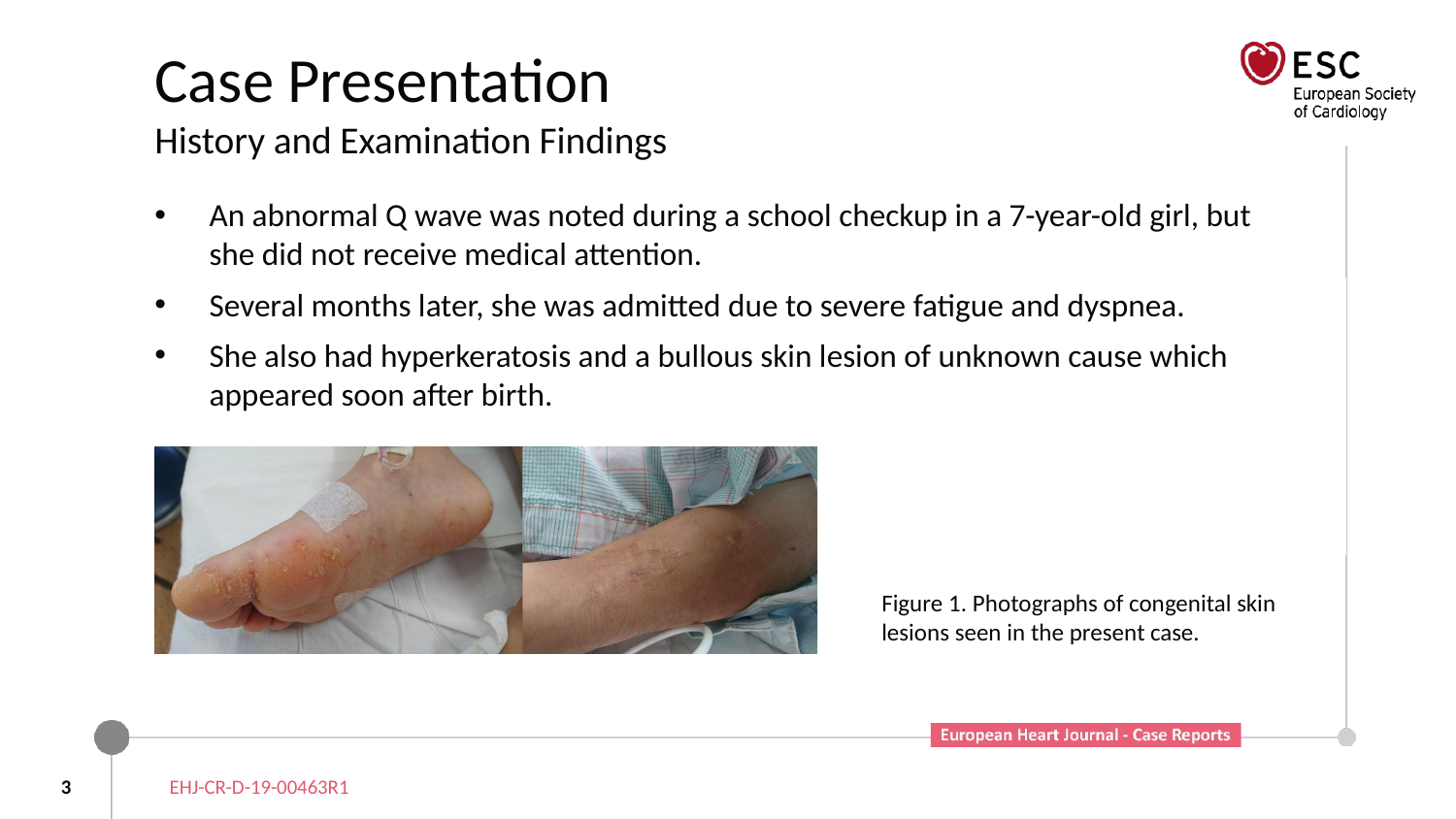

# Case Presentation History and Examination Findings
An abnormal Q wave was noted during a school checkup in a 7-year-old girl, but she did not receive medical attention.
Several months later, she was admitted due to severe fatigue and dyspnea.
She also had hyperkeratosis and a bullous skin lesion of unknown cause which appeared soon after birth.
Figure 1. Photographs of congenital skin lesions seen in the present case.
3
EHJ-CR-D-19-00463R1

## Slide 4
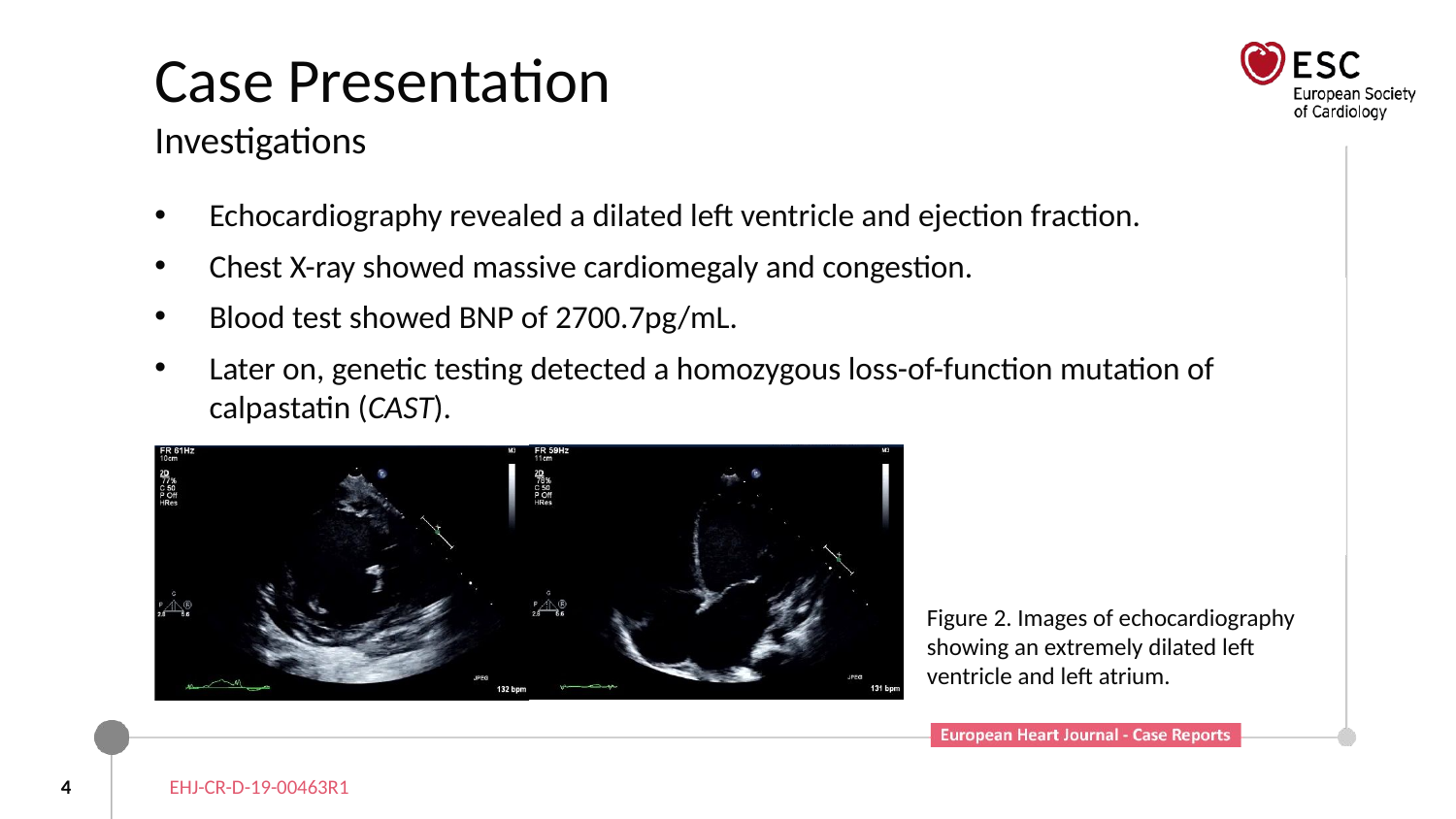

# Case Presentation Investigations
Echocardiography revealed a dilated left ventricle and ejection fraction.
Chest X-ray showed massive cardiomegaly and congestion.
Blood test showed BNP of 2700.7pg/mL.
Later on, genetic testing detected a homozygous loss-of-function mutation of calpastatin (CAST).
Figure 2. Images of echocardiography showing an extremely dilated left ventricle and left atrium.
4
EHJ-CR-D-19-00463R1

## Slide 5
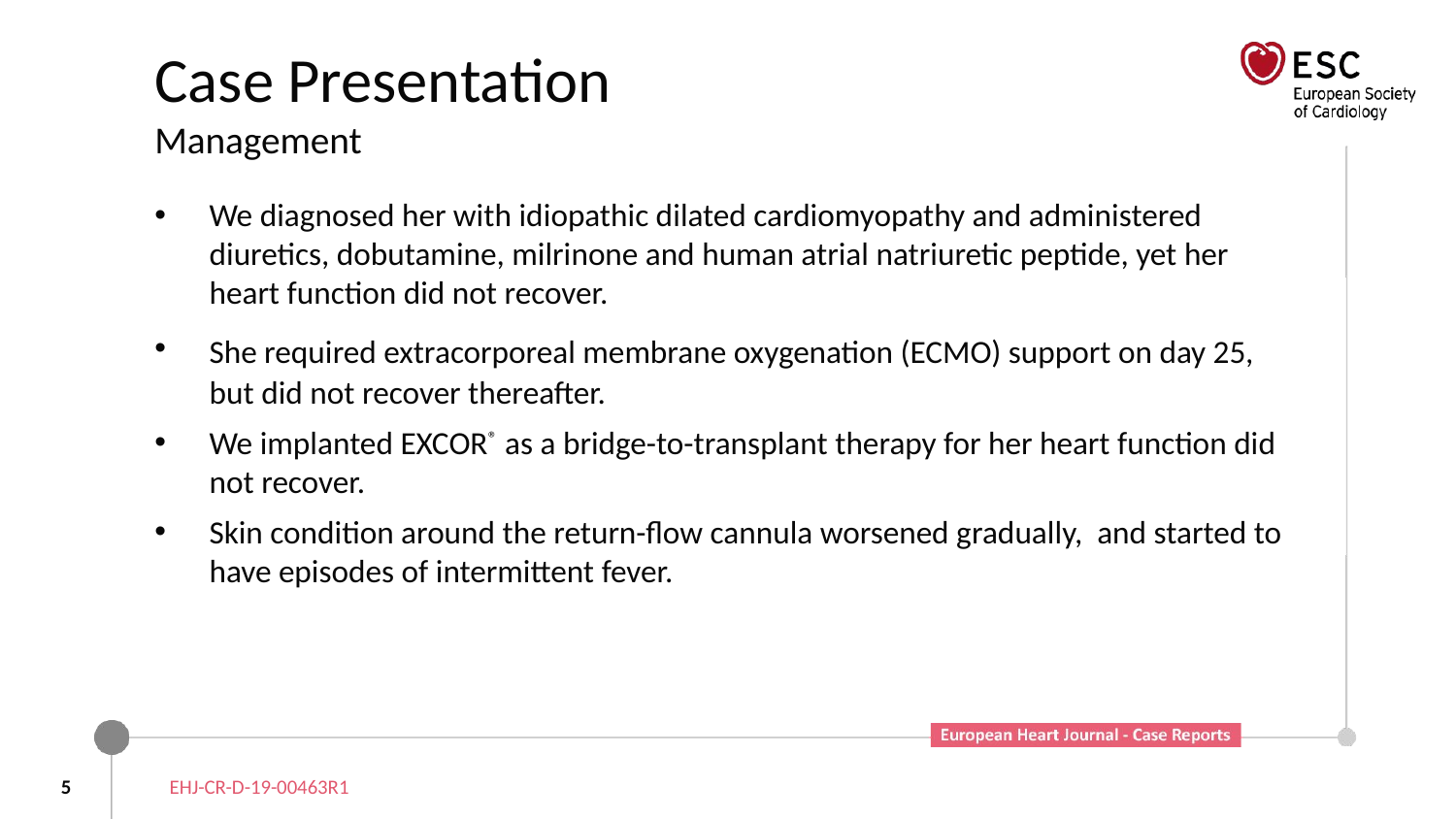

# Case Presentation Management
We diagnosed her with idiopathic dilated cardiomyopathy and administered diuretics, dobutamine, milrinone and human atrial natriuretic peptide, yet her heart function did not recover.
She required extracorporeal membrane oxygenation (ECMO) support on day 25, but did not recover thereafter.
We implanted EXCOR® as a bridge-to-transplant therapy for her heart function did not recover.
Skin condition around the return-flow cannula worsened gradually, and started to have episodes of intermittent fever.
5
EHJ-CR-D-19-00463R1

## Slide 6
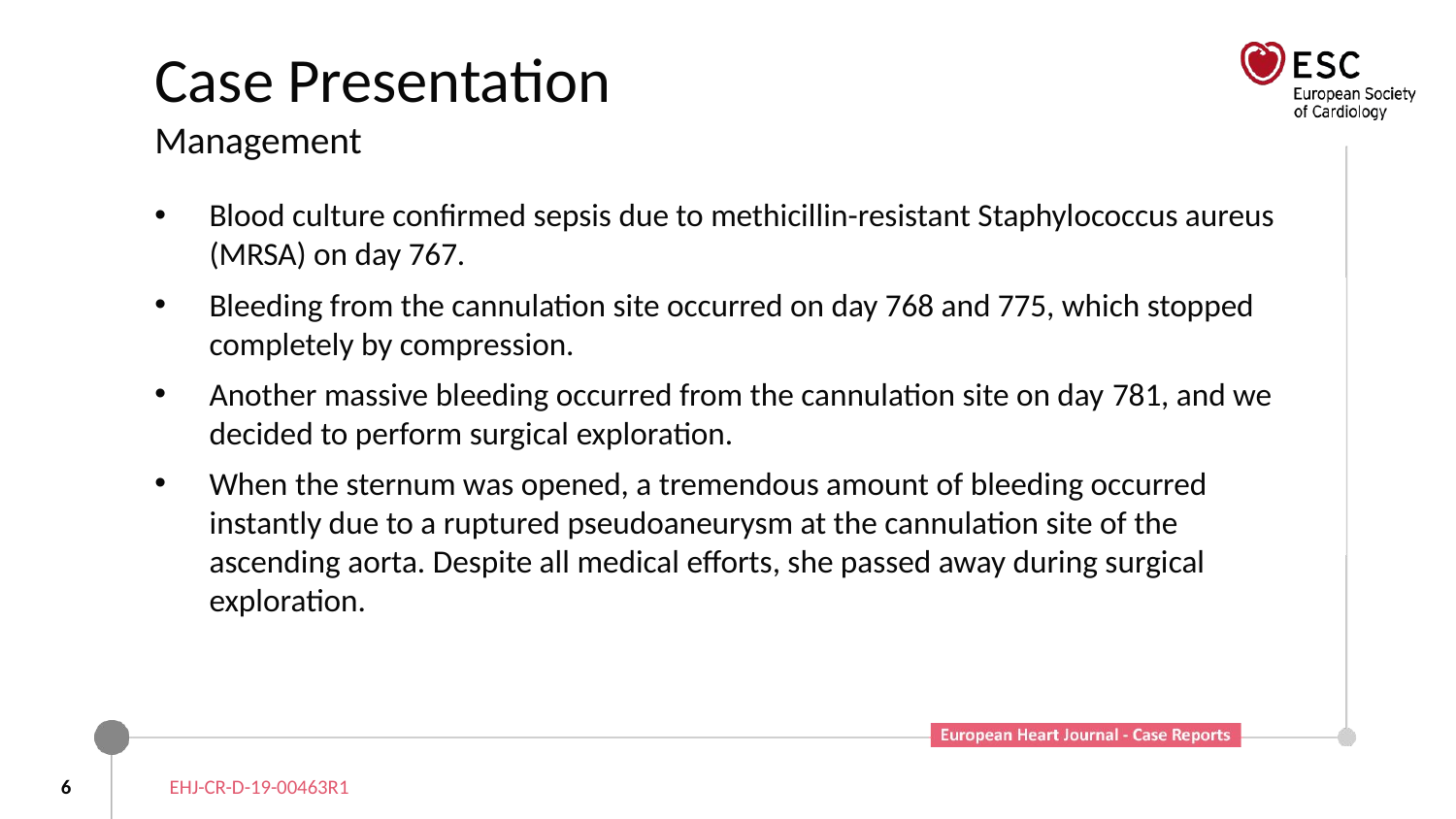

# Case Presentation Management
Blood culture confirmed sepsis due to methicillin-resistant Staphylococcus aureus (MRSA) on day 767.
Bleeding from the cannulation site occurred on day 768 and 775, which stopped completely by compression.
Another massive bleeding occurred from the cannulation site on day 781, and we decided to perform surgical exploration.
When the sternum was opened, a tremendous amount of bleeding occurred instantly due to a ruptured pseudoaneurysm at the cannulation site of the ascending aorta. Despite all medical efforts, she passed away during surgical exploration.
6
EHJ-CR-D-19-00463R1

## Slide 7
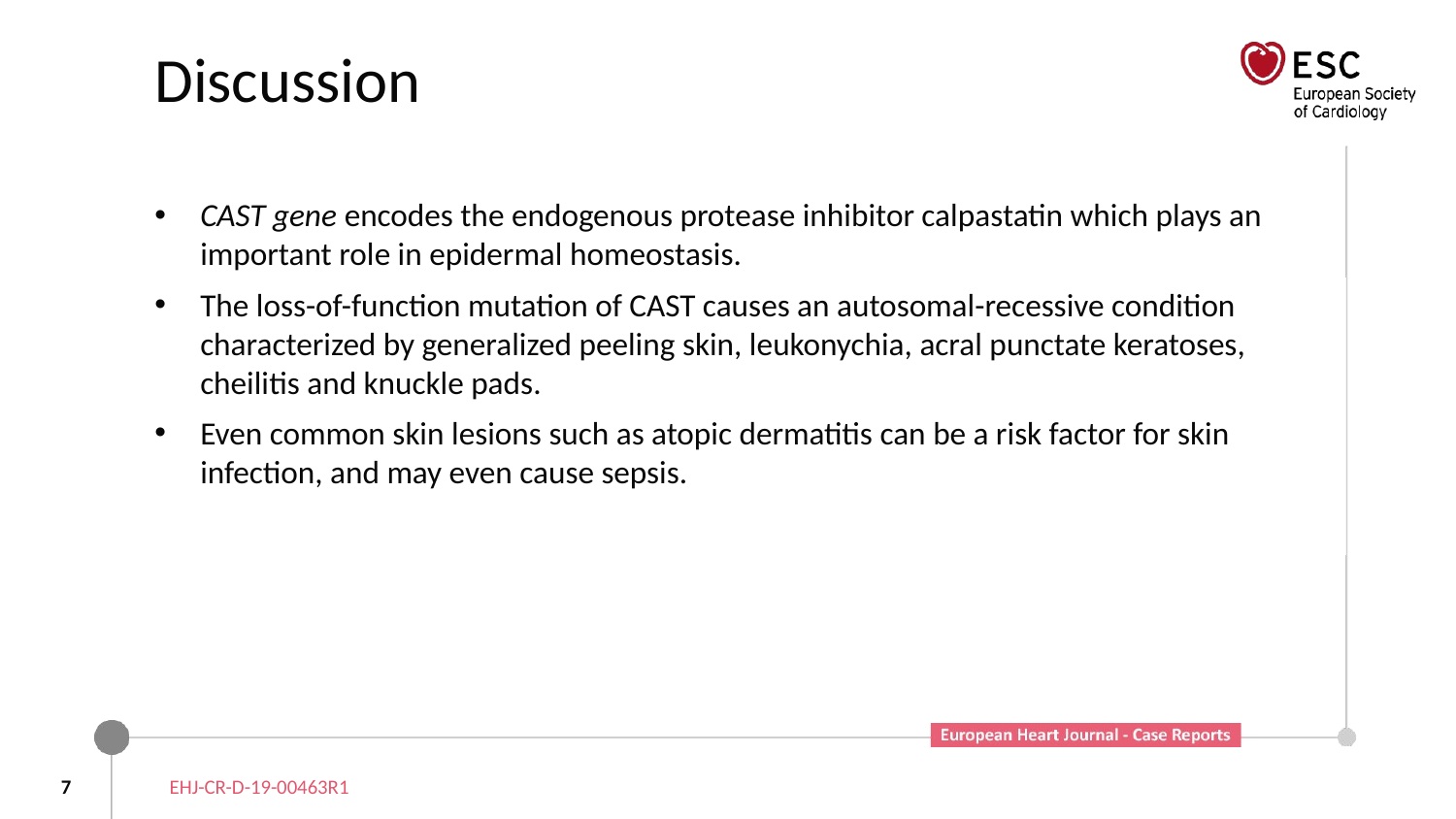

# Discussion
CAST gene encodes the endogenous protease inhibitor calpastatin which plays an important role in epidermal homeostasis.
The loss-of-function mutation of CAST causes an autosomal-recessive condition characterized by generalized peeling skin, leukonychia, acral punctate keratoses, cheilitis and knuckle pads.
Even common skin lesions such as atopic dermatitis can be a risk factor for skin infection, and may even cause sepsis.
7
EHJ-CR-D-19-00463R1

## Slide 8
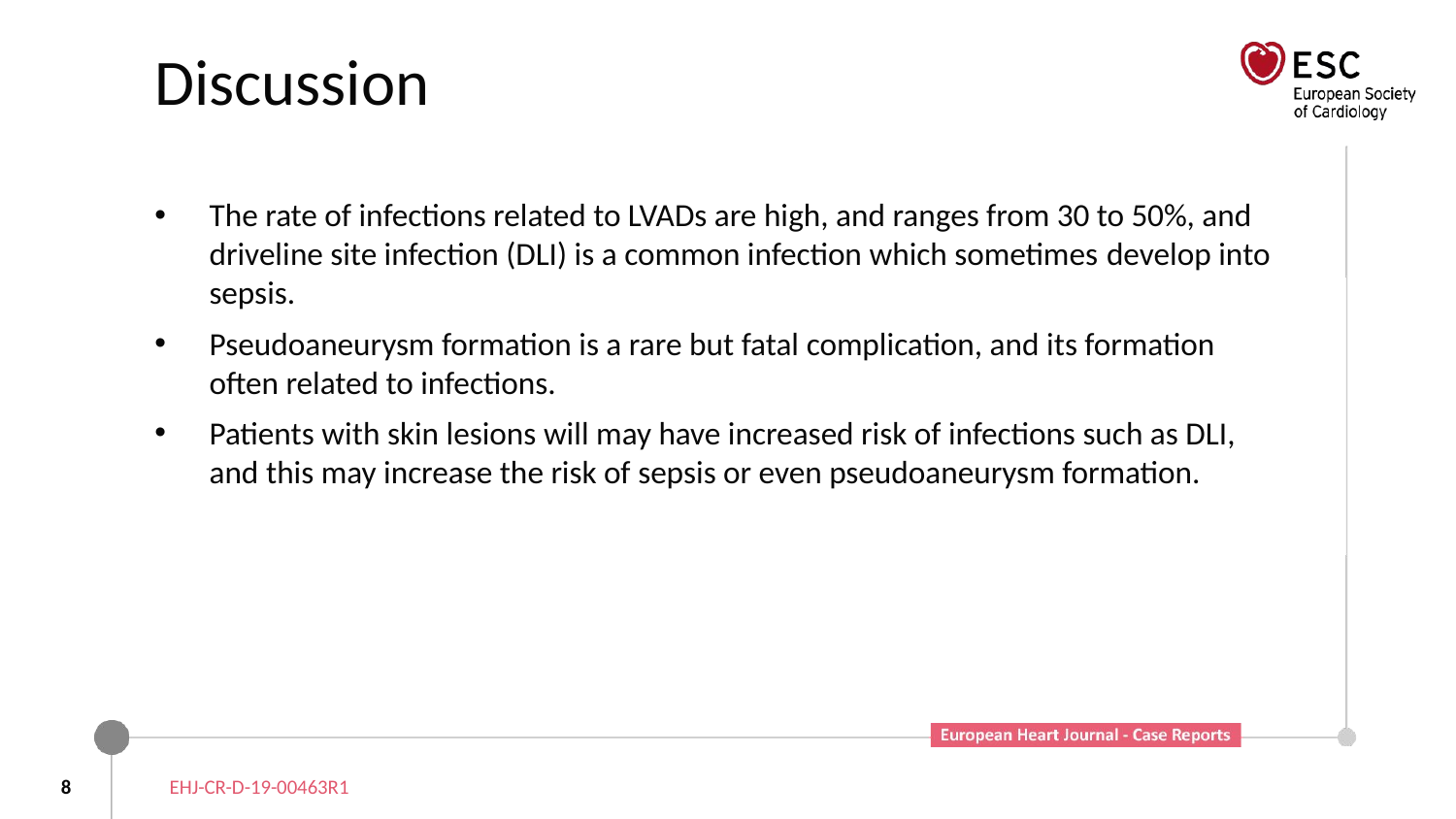

# Discussion
The rate of infections related to LVADs are high, and ranges from 30 to 50%, and driveline site infection (DLI) is a common infection which sometimes develop into sepsis.
Pseudoaneurysm formation is a rare but fatal complication, and its formation often related to infections.
Patients with skin lesions will may have increased risk of infections such as DLI, and this may increase the risk of sepsis or even pseudoaneurysm formation.
8
EHJ-CR-D-19-00463R1

## Slide 9
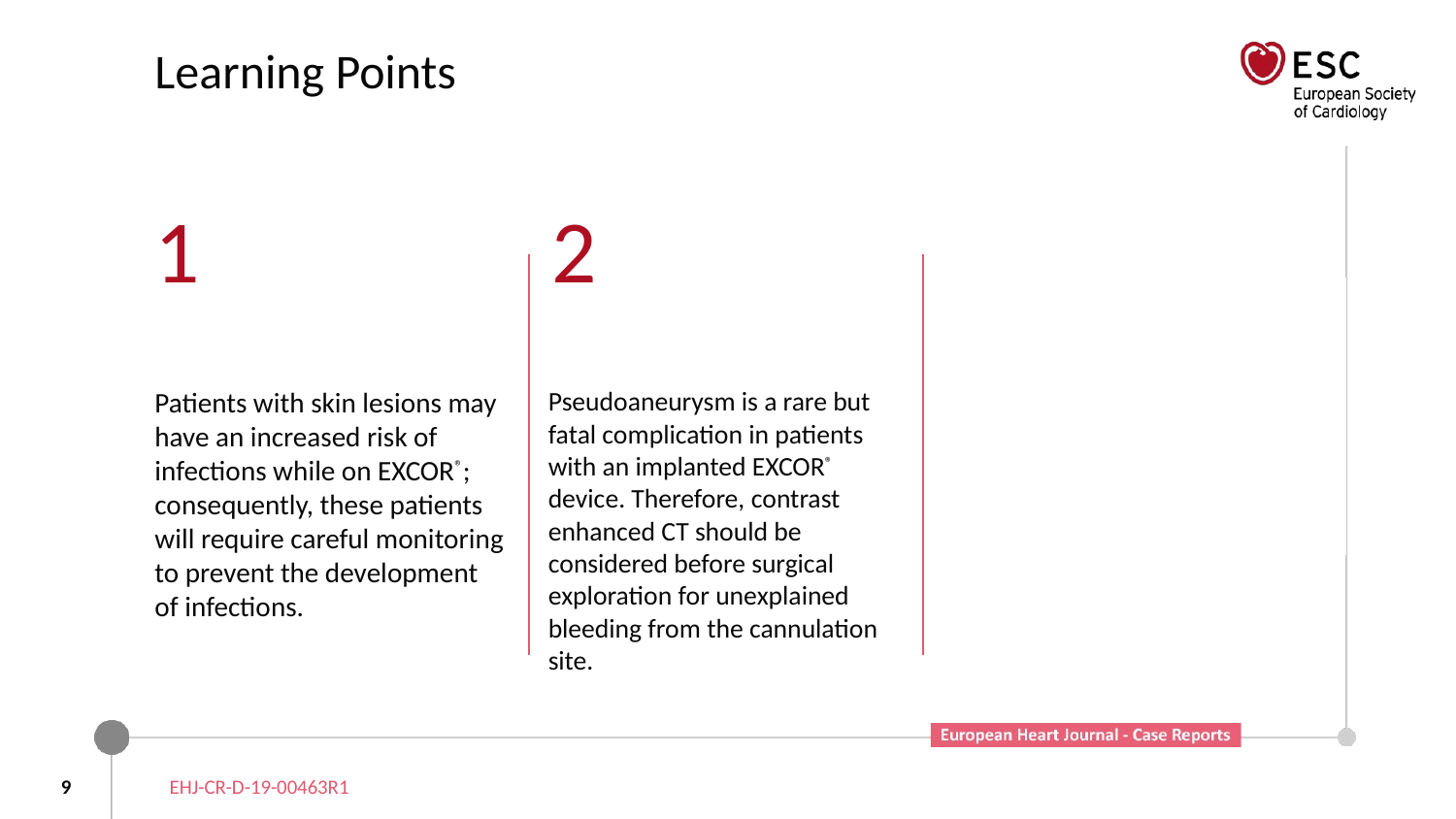

# Learning Points
1
2
Patients with skin lesions may have an increased risk of infections while on EXCOR®; consequently, these patients will require careful monitoring to prevent the development of infections.
Pseudoaneurysm is a rare but fatal complication in patients with an implanted EXCOR® device. Therefore, contrast enhanced CT should be considered before surgical exploration for unexplained bleeding from the cannulation site.
9
EHJ-CR-D-19-00463R1

## Slide 10
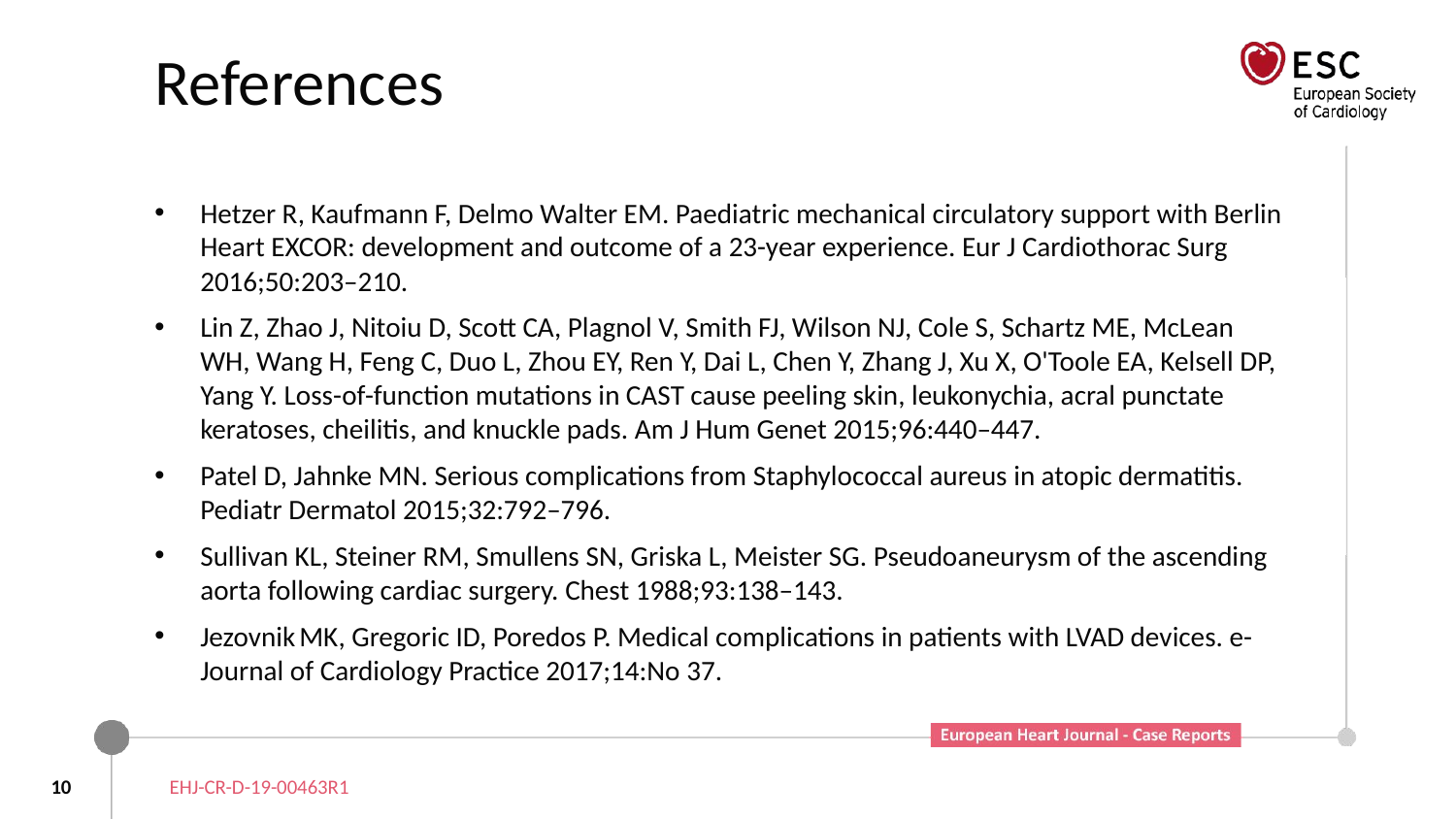

# References
Hetzer R, Kaufmann F, Delmo Walter EM. Paediatric mechanical circulatory support with Berlin Heart EXCOR: development and outcome of a 23-year experience. Eur J Cardiothorac Surg 2016;50:203–210.
Lin Z, Zhao J, Nitoiu D, Scott CA, Plagnol V, Smith FJ, Wilson NJ, Cole S, Schartz ME, McLean WH, Wang H, Feng C, Duo L, Zhou EY, Ren Y, Dai L, Chen Y, Zhang J, Xu X, O'Toole EA, Kelsell DP, Yang Y. Loss-of-function mutations in CAST cause peeling skin, leukonychia, acral punctate keratoses, cheilitis, and knuckle pads. Am J Hum Genet 2015;96:440–447.
Patel D, Jahnke MN. Serious complications from Staphylococcal aureus in atopic dermatitis. Pediatr Dermatol 2015;32:792–796.
Sullivan KL, Steiner RM, Smullens SN, Griska L, Meister SG. Pseudoaneurysm of the ascending aorta following cardiac surgery. Chest 1988;93:138–143.
Jezovnik MK, Gregoric ID, Poredos P. Medical complications in patients with LVAD devices. e-Journal of Cardiology Practice 2017;14:No 37.
10
EHJ-CR-D-19-00463R1
